# Cuttlefish Early Development and Behavior Under Future High CO_2_ Conditions

**DOI:** 10.3389/fphys.2019.00975

**Published:** 2019-07-26

**Authors:** Érica Moura, Marta Pimentel, Catarina P. Santos, Eduardo Sampaio, Maria Rita Pegado, Vanessa Madeira Lopes, Rui Rosa

**Affiliations:** ^1^MARE – Centro de Ciências do Mar e do Ambiente, Laboratório Marítimo da Guia, Faculdade de Ciências da Universidade de Lisboa, Cascais, Portugal; ^2^Department of Collective Behaviour, Max Planck Institute of Animal Behavior, University of Konstanz, Konstanz, Germany; ^3^Centre for the Advanced Study of Collective Behaviour, University of Konstanz, Konstanz, Germany

**Keywords:** ocean acidification, cuttlefish, early life stages, embryogenesis, behavior

## Abstract

The oceanic uptake of carbon dioxide (CO_2_) is increasing and changing the seawater chemistry, a phenomenon known as ocean acidification (OA). Besides the expected physiological impairments, there is an increasing evidence of detrimental OA effects on the behavioral ecology of certain marine taxa, including cephalopods. Within this context, the main goal of this study was to investigate, for the first time, the OA effects (∼1000 μatm; ΔpH = 0.4) in the development and behavioral ecology (namely shelter-seeking, hunting and response to a visual alarm cue) of the common cuttlefish (*Sepia officinalis*) early life stages, throughout the entire embryogenesis until 20 days after hatching. There was no evidence that OA conditions compromised the cuttlefish embryogenesis – namely development time, hatching success, survival rate and biometric data (length, weight and Fulton’s condition index) of newly hatched cuttlefish were similar between the normocapnic and hypercapnic treatments. The present findings also suggest a certain behavioral resilience of the cuttlefish hatchlings toward near-future OA conditions. Shelter-seeking, hunting and response to a visual alarm cue did not show significant differences between treatments. Thus, we argue that cuttlefishes’ nekton-benthic (and active) lifestyle, their adaptability to highly dynamic coastal and estuarine zones, and the already harsh conditions (hypoxia and hypercapnia) inside their eggs provide a degree of phenotypic plasticity that may favor the odds of the recruits in a future acidified ocean. Nonetheless, the interacting effects of multiple stressors should be further addressed, to accurately predict the resilience of this ecologically and economically important species in the oceans of tomorrow.

## Introduction

Over the past centuries, atmospheric carbon dioxide (CO_2_) concentration has been increasing, with a current value of ∼ 415 ppm (NOAA, 2019), being the highest registered in the past 800,000 years. However, due to human dependence on fossil fuels combustion, it is expected to continue rising until 950–1000 ppm (in the higher-emissions scenario – RCP8.5) by 2100 (Rhein et al., 2013).

Oceans absorb about 30% of the CO_2_ present in the atmosphere. This natural process of CO_2_ absorption is driven by the physico-chemical balance between the differences in partial pressure of CO_2_ between the air and the sea surface (Ciais et al., 2013). However, when a higher amount of CO_2_ reacts with seawater, it increases the formation of carbonic acid (H_2_CO_3_), increases the amount of bicarbonate ions (HCO_3_^–^) and reduces the availability of carbonate ions (CO_3_^2–^) (Rhein et al., 2013). These changes cause an increase in the production of hydrogen ions (H^+^) and a subsequent reduction of seawater pH. When this process happens for an extended period it is known as ocean acidification (OA). As CO_2_ levels continue to rise, forecasts indicate that, by the end of this century, the average ocean surface pH will be 0.2–0.4 pH units lower than present values (Rhein et al., 2013). Therefore, this pH drop may represent a serious threat to the health of the world’s ocean ecosystems (Cubasch et al., 2013).

Calcifying organisms, as well as those with minimal physiological buffering capacities (e.g., calcareous sponges, corals and most echinoderms) (Knoll et al., 2007) are expected to be particularly affected by OA. On the other hand, higher resilience is expected from organisms equipped with a more powerful capability to maintain their homeostasis and to compensate for extra and intracellular pH perturbations, such as teleosts and cephalopods (Hu et al., 2015). Nonetheless, an increasing number of studies have been reporting a myriad of OA-related impacts over these mollusks (Rosa et al., 2013; Pimentel et al., 2015, 2016; Spady et al., 2018).

Several studies have already demonstrated, for certain marine taxa, including cephalopods, the detrimental consequences of OA at different behavioral levels, e.g., foraging (Dixson et al., 2015; Pimentel et al., 2016), hunting (Dixson et al., 2015; Pistevos et al., 2017; Spady et al., 2018), predation vulnerability (Dixson et al., 2010; Munday et al., 2014, 2016) and response to olfactory cues (Munday et al., 2009; Dixson et al., 2010). One of the mechanisms pointed for some behavioral disruptions when individuals are exposed to OA is the GABA-A receptor. The GABA-A receptor is the primary inhibitory neurotransmitter in the vertebrate and invertebrate’s brain and, when connected with GABA (γ-aminobutyric acid) in OA conditions, suffers an outflow of Cl^–^ (chloride ions) and/or HCO_3_^–^ from neurons, resulting in depolarization and excitation, i.e., abnormal behaviors (Nilsson et al., 2012; Tresguerres and Hamilton, 2017). However, more research is still needed to better understand how this is underpinned by disruption in brain functions.

Cephalopods are the most neural-developed invertebrates and are considered to have vertebrate-like cognition, underpinned by a well-developed brain, a complex nervous system and sophisticated sensory organs (Fiorito et al., 2015). All cephalopods’ hatchlings have a close-to-optimal central nervous system and most of them are strikingly similar to adults, both in morphology and basic behaviors, such as signaling and camouflage (Boyle, 1987). Additionally, cephalopods present a high level of plasticity to environmental changes (Fiorito et al., 2015; Doubleday et al., 2016), with very effective regulatory and excretory systems to that allow them to tolerate high CO_2_ concentrations over long exposure times (Hu et al., 2015). However, there are differences within the cephalopods’ species, since active pelagic squids show higher sensitivity to elevated *p*CO_2_ when compared with cuttlefish and octopods, which have a nekton-benthic and benthic lifestyles, respectively (Hu et al., 2014). Such differences suggest that different lifestyles and energetic limitations can be a key feature in the ability to mobilize energy resources to fuel acid-base compensatory processes (Hu et al., 2014).

The common cuttlefish (*Sepia officinalis*) is a nekton-benthic species (Reid et al., 2005; Guerra, 2006) with its major activity during the night, spending the day camouflaged on the sand to avoid predators (Boyle, 1987; Reid et al., 2005). Its well-developed and wide visual field (Mäthger et al., 2013) is highly important to its defensive (Chichery and Chanelet, 1976; Boyle, 1987; Reid et al., 2005) and predatory behaviors (Wells, 1958; Messenger, 1968), taking into account that these animals are active predators (Wells, 1958; Messenger, 1968). Besides this, this species has an active lifestyle with high metabolic rates, it is naturally exposed to hypercapnia during its embryonic development (Melzner et al., 2009) and has an elevated tolerance for different environments (Reid et al., 2005; Guerra, 2006).

Previous studies on the impact of OA on cuttlefish have mainly focused on impacts on the cuttlebone (Gutowska et al., 2008, 2010; Dorey et al., 2013; Sigwart et al., 2015), embryonic development (Lacoue-Labarthe et al., 2009; Hu et al., 2011; Dorey et al., 2013; Rosa et al., 2013; Sigwart et al., 2015) and development of newly hatched/juvenile individuals (Gutowska et al., 2008; Lacoue-Labarthe et al., 2009; Hu et al., 2011; Dorey et al., 2013; Sigwart et al., 2015). Regarding the cuttlebone mineralization process, all studies pointed out hypercalcification under high CO_2_ levels (Gutowska et al., 2008; Dorey et al., 2013; Sigwart et al., 2015), but with an irregular CaCO_3_ deposition (Gutowska et al., 2010). The other studies also suggest: (i) an increase in the frequency of premature hatching, (ii) an increase of pre-hatching critical partial pressure (P_crit_ – the point at which the rate of oxygen consumption was no longer maintained independently of ambient *p*O_2_), (iii) a decrease in pre-hatching routine metabolic rate (Rosa et al., 2013), and (iv) decrease of perivitelline fluid pH (Hu et al., 2011). Studies assessing OA impacts on cephalopods’ behavior have so far focused solely on squids (Spady et al., 2014, 2018), while the effects over other cephalopod groups, including cuttlefishes, remain largely unaddressed (Maneja et al., 2011).

In this context, the general objective of the present study was to evaluate, for the first time, a set of developmental and behavioral responses in early developmental stages, by exposing embryos and soon-after hatchlings to OA (ΔpH = 0.4, ∼1000 μatm). To this end, some features as embryonic development time, hatching success, survival rate and biometrics (weight, length and Fulton’s condition index) throughout the embryogenesis and 20 days after hatching (DAH) were analyzed. Moreover, an array of behavioral trials were performed in cuttlefish 15 DAH, to scrutinize the influence of acidification on critical behaviors for early life stages, namely: (1) shelter-seeking – to evaluate preference for a darker or lighter areas; (2) hunting behavior – to assess the amount of prey captured, reaction and successful catch time latency, and capture effectiveness; (3) response to visual alarm cues (ink) – to evaluate the reaction to a visual conspecific-related stimulus (ink), usually used as an alarm cue.

## Materials and Methods

### Exposure of Embryos and Hatchlings to Ocean Acidification

*Sepia officinalis* eggs, at stage I-VI [initial stage of embryogenesis, Naef (1928)] were hand collected during low tide in Mitrena area located in Sado estuary, on the west coast of Portugal (38°47′25.81^″^ N; 8°79′49.34^″^ W). The eggs were transported to Laboratório Marítimo da Guia (MARE-ULisboa) and immediately transferred to an isolated tank for an acclimatization period of 6 days. It is worth noting that the main feature of the Portuguese western coast is the occurrence of coastal upwelling in response to the intensification of northerly winds. Therefore, cuttlefish inhabiting this region are exposed to seasonal variability of seawater carbonate parameters due to the emergence of deepwater masses (Álvarez-Salgado et al., 1997; Borges and Frankignoulle, 2002), and such variability is observed in Table 1.

**TABLE 1:** Seawater carbonate chemistry during the exposure of *Sepia officinalis* to different pH conditions (three replicates for each treatment).

**Parameters**	**Control**	**Acidification**
Temperature (°C)	17.8±0.9	17.9±0.8
Salinity	36±1	36±1
pH	8.05±0.06	7.73±0.07
TA (μmol/kgSW)	2584.7±267.2	2522.0±248.5
*p*CO_2_ (μatm)	461.6±85.1	1016.3±180.7
TCO_2_ (mmol/kgSW)	2316.7±214.2	2406.4±230.7
HCO_3_^–^ (mmol/kgSW)	2101.1±176.8	2266.9±213.6

*Values for *p*CO_2_ were calculated weekly from salinity, temperature, pH total scale (pH) and total alkalinity (TA), using CO2SYS software (Lewis et al., 1998). Values are represented as mean ± standard deviation.*

After the acclimatization period, the eggs were incubated under two *p*CO_2_ treatments (3 replicates each treatment; *N* = 85 eggs per replicate) for ∼65 days (∼45 days during the embryonic development plus 20 days after hatching). The *p*CO_2_ scenarios were chosen to reflect: (a) the annual present pH conditions (*p*CO_2_ ∼ 400 ppm; pH = 8.1) and (b) the near-future expected *p*CO_2_ (*p*CO_2_ ∼1000 ppm; ΔpH = 0.4; based on RCP8.5 projections). The acclimation period took place in a total of six independent experimental life support systems (each tank with 22 L of volume) supplied by natural seawater filtered down to 0.35 μm and through UV radiation, under a semi-closed system. To further assure seawater quality, the experimental tanks were also equipped with mechanical (glass wool) and biological (bioballs matured with nitrifying bacteria) filtration. Temperature was set to 18°C, the average temperature of the spawning season of *S. officinalis* in the western coast of Portugal, and controlled by placing the experimental tanks in water baths connected to chillers (Hailea, Guangdong, China). Room illumination was provided through overhead fluorescent lighting (MASTER TL-D Super 80, 4000 K, 3350 lumen), under a photoperiod of 12 h light: 12 h dark.

During exposure, and on a daily basis, pH, salinity and temperature were monitored manually, as well as hatching and mortality rates. The total alkalinity (TA) was calculated weekly, using the absorbance of water samples measured with an UV spectrophotometer (UV-1800 spectrophotometer, Shimadzu, North America) (Sarazin et al., 1999). Seawater carbonate system speciation was also calculated weekly from salinity, temperature, TA, and pH (total scale) measurements, using CO2SYS software (Lewis et al., 1998) with dissociation constants from Mehrbach et al. (1973) as refitted by Dickson and Millero (1987) (see Table 1). pH was quantified manually with Metrohm pH meter (826 pH mobile, Metrohm, Filderstadt, Germany) connected to a glass electrode (±0.001; Schott IoLine, SI analytics, Mainz, Germany) and calibrated against the seawater buffers Tris–HCl (Tris) and 2-aminopyridine-HCl (AMP) (Mare, Liège, Belgium) according to Dickson et al. (2007). The experimental pH levels were adjusted automatically, via solenoid valves controlled by an automated system (Profilux 3, Kaiserslautern, Germany) connected to individual pH probes (Blueline 25 pH, SCHOTT Instruments, Mainz, Germany). Profilux pH hysteresis was set at ±0.05 margins (lower limit of the system), to minimize the degree of *p*CO_2_ variation inherent to simulated hypercapnic treatments (as observed in Table 1) and the respective repercussions (see Jarrold and Munday, 2019 and references therein). The pH of natural seawater was reduced by the injection of a certified CO_2_ gas mixture (Air Liquide, Miraflores, Algés, Portugal), via air stones, and balanced positively through aeration with CO_2_-filtered air (using soda lime, Sigma-Aldrich, St. Louis, MO, United States). Salinity was measured using a refractometer (V2Refractometer, TMC, Iberia, Portugal) and maintained (∼35) by adding more seawater. Ammonia, nitrites and nitrates levels were monitored using colorimetric tests (Tropical Marine Centre, United Kingdom) and maintained below detectable levels, lower than 0.5, 0.2, and 80 mg/L (Fiorito et al., 2015), respectively. The cuttlefish hatchlings were fed *ad libitum* with frozen brine shrimp enriched with spirulina and the uneaten food was removed at the end of each day.

### Hatchlings’ Biometrics

Dorsal mantle length (DML), total body length (TBL) and total body weight (TBW) were measured in all individuals with 20 DAH. Both mantle and total length were measured through image analysis and the TBW was registered with an analytical scale. Fulton’s Condition Index (*K*) was calculated according to Fiorito et al. (2015), as follows:

$$K=\frac{T\,B\,W}{D\,M\,L^{3}}\times 100$$

These measurements were performed in two different groups, due to the difference in development time upon collection. Hatching and mortalities were monitored daily to further calculate development time, hatching success and survival rate. At the end of the experiment, individuals were anesthetized and euthanized, according to the Guidelines for the Care and Welfare of Cephalopods in Research (Fiorito et al., 2015), and preserved for future biochemical analysis.

### Behavioral Analysis

The behavioral tests performed were: (1) shelter-seeking, (2) hunting behavior and (3) visual detection of a conspecific-related stimulus. All tests were performed with hatchlings 15–20 DAH. Individuals participating in the first two behavioral tests were trialed in different days. Individual cuttlefish were carefully transferred from its holding tanks to the arena of each test and all arenas were designed according with the specific needs of each test. All performed tests, described below, were performed following a 10 min acclimatization period (Darmaillacq et al., 2004; O’Brien et al., 2016, 2017) and recorded with a Canon LEGRIA HF R56 camera. At the end of each test, individuals were returned to their holding tanks and testing arenas were cleaned between trials.

#### Shelter-Seeking

Shelter-seeking tests ran in a rectangular arena (area = 145 cm^2^; volume = 4000 mL) with dark walls and a transparent bottom. Half of the arena was topped with a dark and opaque cover (14.5 cm Black + 14.5 cm Opaque), as to provide a fully shaded area, while a light and semi-translucid cover was placed over the other half as to produce an area uniformly illuminated by a diffuse light, placed circa 50 cm above the arena. In this arena, a neutral area (4 cm) was assigned and adjusted according to Jutfelt et al. (2017). Each cuttlefish was randomly and gently introduced through a small entryway in the middle of the upper zone. Following acclimatization period, activity was recorded during 15 min. using a camera placed underneath the arena. An individual was assumed to have a light or dark preference only if it left the neutral area and spent ≥70% of the total test time (≥630 s) in one of the chosen areas. If both these criteria were not met, the individual was assumed to have no preference for either light or dark conditions. The sides corresponding to the lighter and darker areas were switched randomly between trials to prevent lateralization-associated bias.

#### Hunting

An opaque arena (area = 80 cm^2^; volume = 500 mL) with a small sand shelter, to minimize stress, was used to observe the hatchlings hunting behavior. A single random cuttlefish was gently placed in the arena and the test was performed for 10 min, after the acclimatization period and after the introduction of the prey. To each cuttlefish 5 prey items (*Gammarus* sp.) were introduced through a tube present at a corner of the tank and available to hunt during the test, since a preliminary test revealed that 5 was the maximum number of prey that one individual could consume within the test period. The attack latency time was accounted between the reaction to the prey and its catch (successful attack). A lamp was placed 30 cm above the arena to ensure enough and equally distributed lighting and the cuttlefish activity was recorded with a camera at the same high. A total of 28 cuttlefish and 140 prey were used for this test.

#### Visual Detection of Conspecific Visual Stimulus – Ink

The purpose of this test was to evaluate the response to the visual component of an ink stimulus, used as a defense mechanism and perceived as an alarm cue in cephalopod species (Gilly and Lucero, 1992; Boal and Golden, 1999; Bush and Robison, 2007; Wood et al., 2008; Mezrai et al., 2018). This was tested using a round glass arena (area = 100 cm^2^, volume = 500 mL), with a central glass compartment that allowed the visual display of the cue (commercial cuttlefish ink) whilst blocking the chemically mediated cue component. The test started with the introduction of the cue, that took place from above through an opaque tube fixed above the cue-compartment. To avoid disturbance to the test, both the perimeter and the top of the arena were fully covered in an opaque overlay from which the cue-introducing tube was placed. Additionally, a sham-test, in which clear seawater was introduced instead of the ink, was performed after the acclimatization and 5 min. prior to the stimulus introduction, in order to control for the presence of other factors. A lamp was placed above the arena and a camera was placed below to record the cuttlefish’s activity, after the acclimatization period, including the sham-test and the reaction to the ink. Here, a total of 28 individuals was used and their reactions were divided into four classes (0 = no perceived reaction; 1 = increase of ventilation and/or branchial movements; 2 = color changes and/or cessation of swimming; 3 = escape, attack and/or dorsal arms raised) adapted from Wood et al. (2008).

### Data Analysis

All image and video footage were analyzed with specific programs, i.e., ImageJ was used to obtain biometric data (length measures) from photography and BORIS software (Behavioral Observation Research Interactive Software v.6.0.5 – 2018-01-29) was used to analyze all video data. While using BORIS software, specific commands were defined considering the specificities of each test. For the shelter-seeking test, four commands were defined to register the time spent in each area: (a) start of test, (b) entrance into the lighted area, (c) entrance into the shaded area, and (d) entrance into the neutral area. For the hunting behavior test, four commands were defined to acquire the timings and attack effectiveness of the individuals: (a) prey introduction, (b) reaction to the prey, (c) attack, and (d) catch. For the visual detection test, five commands were defined: (a) reaction, (b) no perceived reaction, (c) reaction 1 (increase of ventilation and/or branchial movements), (d) reaction 2 (color changes and/or cessation of swimming) and (e) reaction 3 (escape, attack and/or dorsal arms raised).

After data visualization, statistical analyses of the defined variables were performed with RStudio Software (Version 1.1.456 – © 2009–2018 RStudio, Inc.). All Generalized Linear Models (GLM) were performed with pH as factor. For all the variables analyzed, replicates and hatching date (considers the difference in development time upon collection) were first included in generalized linear mixed models (GLMM) as random effect, to account for potential variability in the experimental design and for the dependency within these factors. Random effects were removed from the models whenever the amount of variation explained was lower than 5%. The best model for each output was chosen according to the calculation of Akaike Information Criterion (AIC), i.e., the best model was the one that featured the smallest AIC. The GLMM with Gaussian family was used to analyze weight (with hatching date as a random factor) and DML (with replicates as a random factor). The same model with Gamma family and log link function was used to analyze the Fulton’s index (with replicates and hatching date as random factors). The GLM with Gaussian family was performed to analyze TBL.

The reaction and catch time in the hunting behavior test were analyzed with the Gamma family and inverse link function. The Binomial family of distribution was used to analyze the shelter-seeking test (choice/no choice and black/white) and the visual detection test for reaction variable. Count data were analyzed with the Poisson family, i.e., development time, hatching success, hatchlings survival (within identity as a link function) and the successful attacks observed in the hunting behavior test. To analyze the type of reaction in the visual detection test, a multinomial logistic regression model was performed with the four classes in test, mentioned above. All statistical differences were considered when *p*-value < α with α = 0.05.

## Results

### Development Time, Survival and Hatchlings’ Biometrics

**FIGURE 1:**
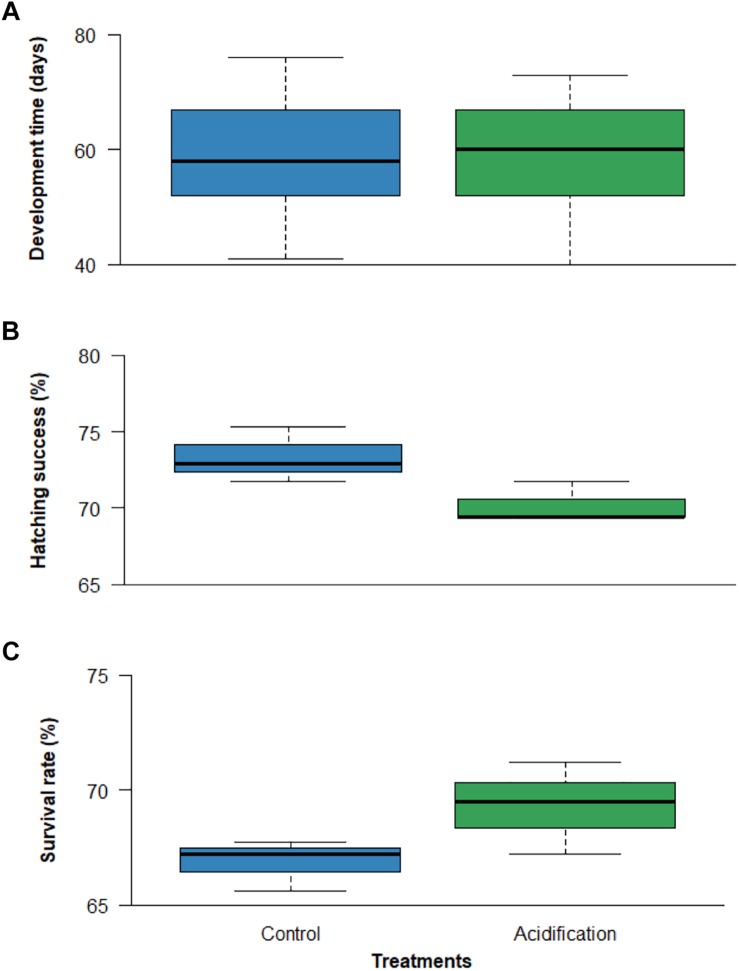
Effect of ocean acidification (ΔpH = 0.4) on: **(A)** development time, **(B)** hatching success and **(C)** survival rate [20 days after hatching] of the common cuttlefish *Sepia officinalis*. Boxplots illustrate median, upper and lower quartile, and inter-quartile range.

Development time was similar in both treatments (∼ 59 ± 9 days), i.e., there was no significant effect under OA (Figure 1; *p* > 0.05; GLM, Poisson family, more details in Supplementary Table 1). Likewise, neither hatching success (73.33 ± 1.80% under normocapnia and 70.20 ± 1.36% under hypercapnia) nor survival rate after 20 DAH (66.86 ± 1.10% and 69.30 ± 1.99%, under normocapnia and hypercapnia, respectively) were significantly affected by high CO_2_ treatment (Figure 1; *p* > 0.05; GLM, Poisson family, see more statistical details in Supplementary Table 1).

**FIGURE 2:**
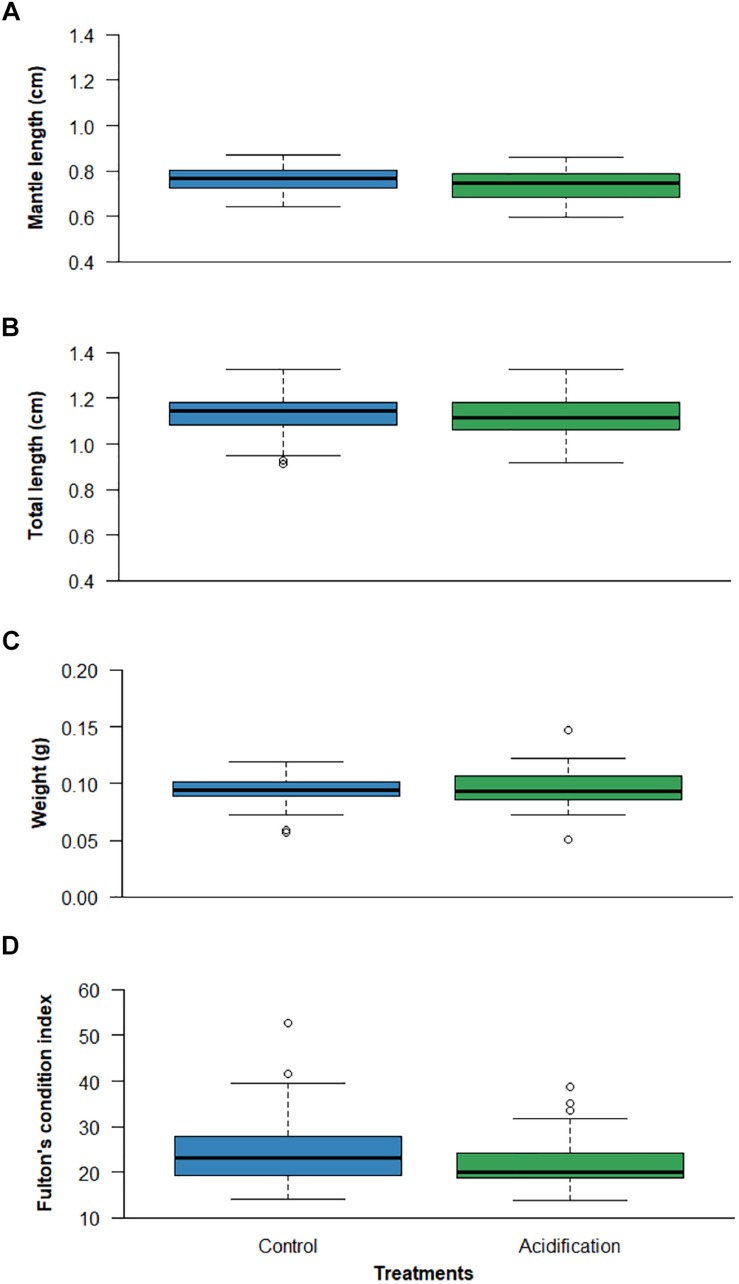
Effect of ocean acidification (ΔpH = 0.4) on: **(A)** dorsal mantle length (DML), **(B)** total body length (TBL), **(C)** total body weight (TBW) and **(D)** Fulton’s condition index (*K*) of the common cuttlefish *Sepia officinalis*. Boxplots illustrate median, upper and lower quartile, and inter-quartile range. Circles indicate individual outliers outside the inter-quartile range.

Ocean acidification effects on the DML (Figure 2; *p* > 0.05; GLMM, Gaussian family, analysis in Supplementary Table 2.), TBL (Figure 2; *p* > 0.05; GLM, Gaussian family, analysis in Supplementary Table 3), TBW (Figure 2; *p* > 0.05; GLMM, Gaussian family, analysis in Supplementary Table 2) and Fulton’s index (*K*) (Figure 2; *p* > 0.05; GLMM, Gamma family, analysis in Supplementary Table 4) were also not statistically significant.

### Behavioral Responses

**FIGURE 3:**
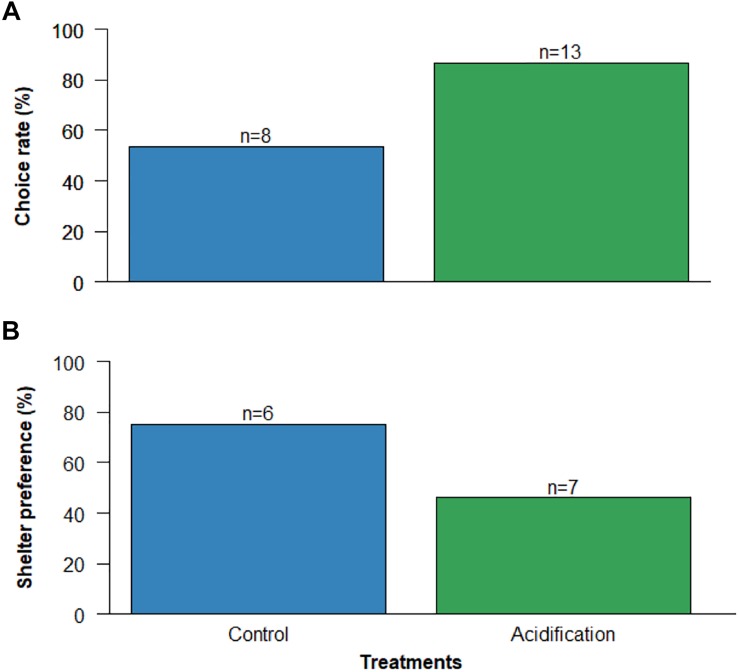
Effect of ocean acidification (ΔpH = 0.4) on the shelter-seeking behavior of the common cuttlefish *Sepia officinalis* with 15–20 DAH: **(A)** capacity to make a choice and **(B)** preference for a darker shelter. Values represent the number of individuals in each treatment who made the respective choice.

No significant differences were found between treatments in the choice rate of shelter, nor in the light/shade preference in the shelter-seeking test (Figure 3; *p* > 0.05; GLM, Binomial family, analysis in Supplementary Table 1).

**FIGURE 4:**
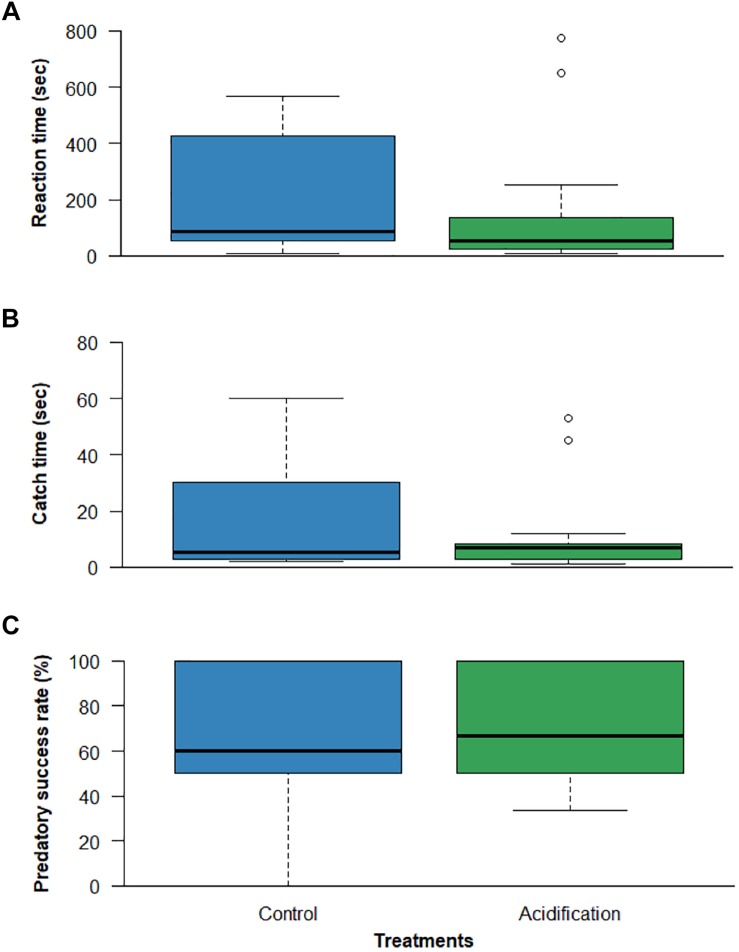
Effect of ocean acidification (ΔpH = 0.4) on: **(A)** time of reaction to the prey, **(B)** time to catch the prey, and **(C)** predatory success of the common cuttlefish *Sepia officinalis* with 15–20 DAH. Boxplots illustrate median, upper and lower quartile, and inter-quartile range. Circles indicate individual outliers outside the inter-quartile range.

Likewise, no significant differences were observed regarding to their hunting behavior (namely in the reaction to prey and in the attack duration) between control and OA treatments (Figure 4; *p* > 0.05; GLM, Gamma family, analysis in Supplementary Table 3), as well as for predatory success rate (Figure 4; *p* > 0.05; GLM, Poisson family, more details in Supplementary Table 1).

**FIGURE 5:**
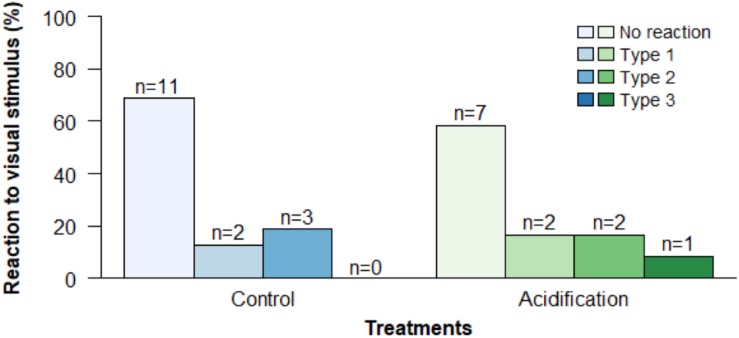
Effect of ocean acidification (ΔpH = 0.4) on the reaction to a conspecific visual stimulus of the common cuttlefish *Sepia officinalis* with 15–20 DAH. Values represent the number of individuals in each treatment who made the respective choice.

Similarly, the visual detection of conspecific visual stimulus (ink) was also not significantly affected by the experimental CO_2_ treatments (Figure 5; *p* > 0.05; GLM, Binomial family for the reaction/no perceived reaction and multinomial logistic regression for the type of reaction, both analyses can be found in Supplementary Table 1). A large proportion of individuals had no perceived reaction to the stimulus, 68.75% under normocapnia and 58.33% under hypercapnia. Noteworthy, 8.33% of the individuals exposed to hypercapnia presented the more severe type of reaction – type 3 (escape, attack and/or dorsal arms raised), whereas no individuals under normocapnia showed this type.

## Discussion

The effects of OA in cephalopod early stages are still fairly unknown. However, some studies suggest that OA does not impair normal embryonic development (Dorey et al., 2013; Rosa et al., 2013), survival rates (Dorey et al., 2013) or body size (Lacoue-Labarthe et al., 2009; Hu et al., 2011; Dorey et al., 2013; Rosa et al., 2013; Sigwart et al., 2015). Accordingly, the results here presented suggest that near-future CO_2_ did not elicit major impacts on the development time, survival rates and size of *S. officinalis* early stages. The abiotic conditions inside cuttlefish eggs have already been characterized as stressful conditions - with high levels of *p*CO_2_ and HCO_3_^–^, and low pH (Melzner et al., 2009; Rosa et al., 2013), a consequence of increasing energy expenditure during egg development and swelling (Lacoue-Labarthe et al., 2009; Dorey et al., 2013). Ocean acidification will amplify these already hypercapnic conditions inside the cuttlefish eggs (Hu et al., 2011; Dorey et al., 2013; Rosa et al., 2013), as the water from the outside environment enters (in this case water with high levels of *p*CO_2_) into the hypertonic perivitelline fluid eggs (Hu et al., 2011; Dorey et al., 2013). Giving these embryonic conditions, cuttlefish hatchlings may be consequently more adapted to develop in the future ocean pH conditions.

The common cuttlefish is a species that usually stays hidden in the sand during daytime, to avoid predation (Boyle, 1987; Reid et al., 2005), registering higher activity during the night (Guerra, 2006). Thus, it would be expected that a higher percentage of individuals would choose the shadow side of the shelter arena under control conditions, because of their preference of dark over light environment (Guerra, 2006). However, a high percentage of animals did not make a choice related with shelter dark/bright under control conditions (46.67%). This may be explained by the fact that these organisms use camouflage as their primary line of defense (Boyle, 1987) and, thus, potentially do not prioritize choosing between different light intensity. Another potential explanation can be the lack of enough lightening contrast, which may have made these animals “comfortable” on the neutral area and in the bright side, removing the necessity to move to a safer option. Further studies focusing on camouflage success should be addressed to better understand these results. These mollusks are active predators (Wells, 1958; Messenger, 1968), which was demonstrated by the short time between the first reaction to prey and the time to effectively catch it (control ∼17 s and acidification ∼12 s). The present findings support those obtained by Maneja et al. (2011), who also showed that cuttlefish early stages do not seem to be affected by near-future OA. However, Spady et al. (2018) found the opposite results in squids, with a decrease of hunting behavior (increase of attack latency in both species – bigfin reef squid and pygmy squid – and reduction in the proportion of individuals who attacked their prey in pygmy squid), in the animals exposed to an acidified environment. These findings support the claim of differences insensitivity to elevated *p*CO_2_ between pelagic (squids) and nekton-benthic/benthic species (cuttlefish and octopods). When compared with cuttlefishes, squids have more difficulty in reallocating energy toward compensatory processes since they have a lifestyle at the edge of energetic limits due to their high locomotory costs (O’dor and Webber, 1986). Therefore, squids showed more sensitivity when exposed to changing environments (Rosa and Seibel, 2008; Hu et al., 2014) than the nekton-benthic/benthic species that have a slower lifestyle and a better pH buffering/regulatory mechanisms and thus, more resilient (Gutowska et al., 2008; Dorey et al., 2013; Hu et al., 2014). In the visual stimulus test, responses with a higher severity level, e.g., reaction type 3, in the animals from control scenario were expected, as it was observed by Wood et al. (2008) with Caribbean reef squid. However, a high percentage of individuals had no perceivable reactions to the ink stimulus, either in control and acidification treatments. Usually, more severe behaviors are also more visible reactions, which may have a higher effective role in warning their conspecifics about the danger nearby. The different results obtained in this study and those obtained by Wood et al. (2008) may be related with the different lifestyles (pelagic and nekton-benthic/benthic species) and with the life-stage of the animals used in these studies, thus early stage animals may prefer to direct their energetic reserves to grow, instead of defensive behaviors without effective acting, i.e., the ink eject effect is smaller in youngers (smaller size) than in adults (bigger size, more ink). Thus, younglings may opt for defensive behaviors that save more energy. Behavioral freeze is known as cuttlefish response toward certain threats (Bedore et al., 2015), which could account for at least some of non-perceived reacting animals. In this context, a more specialized approach focusing on this response would be necessary. Yet, further research must be conducted, especially at the neurological level, to corroborate the lack of behavioral responses reported here, and to understand how changes in other behaviors are underpinned by OA-related disruption in brain functions.

In general, OA affects survival, fitness and behavioral patterns in many marine organisms (Kaplan et al., 2013; Pimentel et al., 2014, 2016; Dixson et al., 2015). Nevertheless, the present study showed that future CO_2_ levels might not elicit significant changes on the developmental and behavioral responses during the early ontogeny (embryos and hatchlings) of the common cuttlefish *S. officinalis*. Such findings are likely related with their nekton-benthic (and active) lifestyle, their adaptability to highly dynamic coastal and estuarine zones, and the already harsh conditions (hypoxia and hypercapnia) inside their eggs, which may favor the odds of the common cuttlefish recruits to endure the future acidified ocean through a possible pre-adaptation to these adverse conditions (Melzner et al., 2009; Hu, 2016). Gutowska et al. (2008) also supported this prediction, showing that *S. officinalis* did not exhibit sensitivity to elevated CO_2_ levels within the range of concentrations that elicits a negative response in most other invertebrates (e.g., corals and bivalves). One of the greatest issues caused by OA is the dissolution of calcium carbonate minerals, affecting some taxa with CaCO_3_ structures [e.g., (Fabry et al., 2008; Stocker et al., 2013)], such as corals [e.g., (Erez et al., 2011; Camp et al., 2017)] and mollusks [e.g., (Kurihara et al., 2007; Talmage and Gobler, 2010; Maneja et al., 2011; Kaplan et al., 2013)]. On the other hand, there are some species that under OA are able to maintain or even increase their calcifying structures, like the cephalopod *S. officinalis* (Gutowska et al., 2008, 2010; Dorey et al., 2013). In some calcifying organisms, this hypercalcification phenomenon appears to be an energy demanding process and the associated energetic trade-offs under acidification affect the organism’s normal growth rates (Stumpp et al., 2011). Yet, it is still not fully understood what the consequences (e.g., metabolic and behavioral level) of this phenomenon on these organisms are.

However, this species showed to be quite resilient to near-future OA. Additionally to the characteristics already mentioned, its short life cycle (1–2 years) may enhance the chance for evolutionary adaptation (Hu, 2016) and detrimental consequences may only turn visible in a very high CO_2_ environment or in combination with other climate change factors such as temperature (Lacoue-Labarthe et al., 2009; Rosa et al., 2013), hypoxia (Rosa et al., 2013), and/or with exposition to contaminants. Nevertheless, the shallow-water environments that *S. officinalis* occupies are particularly susceptible to anthropogenic pressures and climate-change stressors, which makes the study of cumulative effects (i.e., of multiple stressors) of paramount importance to accurately predict how this ecologically and economically important species will fare in the future.

## Data Availability

The raw data supporting the conclusions of this manuscript will be made available by the authors, without undue reservation, to any qualified researcher.

## Ethics Statement

Research was conducted under approval of Faculdade de Ciências da Universidade de Lisboa animal welfare body (ORBEA) and Direção-Geral de Alimentação e Veterinária (DGAV) in accordance with the requirements imposed by the Directive 2010/63/EU of the European Parliament and of the Council of 22 September 2010 on the protection of animals used for scientific purposes.

## Author Contributions

ÉM, RR, MP, CS, ES, and VL designed the experiment. ÉM, MP, CS, and MRP performed the experiment. ÉM, RR, MP, CS, and ES analyzed the data. All authors contributed to the writing of the manuscript.

## Conflict of Interest Statement

The authors declare that the research was conducted in the absence of any commercial or financial relationships that could be construed as a potential conflict of interest.
