# Supplementary material for: Cuttlefish Early Development and Behavior Under Future High CO2 Conditions
Source: Front Physiol. 2019 Jul 26;10:975. doi: 10.3389/fphys.2019.00975 (PMC6676914; doi:10.3389/fphys.2019.00975)
Supplement: Supplementary file 1 [file Table_1.docx]

**Supplemental Table 1.** Results of the statistical models applied for analysis of ocean acidification effects in *Sepia officinalis*. Codes: *** when p-value < 0; ** when p-value < 0.001; * when p-value < 0.05; . when p-value < 0.1.

|  | **Model** | **Fixed effects** | **Terms** | **Estimate** | **Std. error** | **z value** | **Pr(>\|z\|)** |  |
| --- | --- | --- | --- | --- | --- | --- | --- | --- |
| **Hatching success** | GLM, family=Poisson AIC=39.826 | Final number eggs~pH | (Intercept) | -80.866 | 0.073 | -1105.846 | < 0.001 | *** |
|  |  |  | pH | -0.044 | 0.105 | -0.418 | 0.676 |  |
| **Survival** | GLM, family=Poisson, link=identity AIC=37.580 | Final number hatchlings~pH | (Intercept) | -20.640 | 3.726 | -5.539 | < 0.001 | *** |
|  |  |  | pH | 2.334 | 5.260 | 0.444 | 0.657 |  |
| **DED** | GLM, family=Poisson AIC=2610.600 | Time of development~pH | (Intercept) | 4.075 | 0.010 | 425.098 | < 0.001 | *** |
|  |  |  | pH | -0.003 | 0.014 | -0.211 | 0.833 |  |
| **Successful attacks** | GLM, family=Poisson AIC=83.246 | Successful attacks~pH | (Intercept) | 0.588 | 0.192 | 3.054 | 0.002 | ** |
|  |  |  | pH | -0.108 | 0.291 | -0.372 | 0.710 |  |
| **Shelter choice** | GLM, family=Binomial AIC=36.508 | Choice/No choice~pH | (Intercept) | 0.133 | 0.517 | 0.258 | 0.796 |  |
|  |  |  | pH | 1.738 | 0.919 | 1.891 | 0.059 | . |
| **Shelter preference** | GLM, family=Binomial AIC=30.942 | Darker/Bright~pH | (Intercept) | -1.099 | 0.816 | -1.346 | 0.178 |  |
|  |  |  | pH | 1.253 | 0.988 | 1.268 | 0.205 |  |
| **Visual reaction** | GLM, family=Binomial AIC=40.175 | Response to stimulus~pH | (Intercept) | -0.788 | 0.539 | -1.462 | 0.144 |  |
|  |  |  | pH | 0.452 | 0.796 | 0.568 | 0.570 |  |
| **Visual type of reaction** | Multinomial Logistic Regression AIC=44.333 | pH~Kind of reaction | (Intercept) | 0.452 | 0.483 | 0.936 | 0.349 |  |
|  |  |  | Reaction 1 | -0.452 | 1.111 | -0.407 | 0.684 |  |
|  |  |  | Reaction 2 | -0.046 | 1.033 | -0.045 | 0.964 |  |
|  |  |  | Reaction 3 | -8.848 | 66.572 | -0.133 | 0.894 |  |

**Supplemental Table 2.** Results of the statistical models applied for analysis of ocean acidification effects in *Sepia officinalis*. Codes: *** when p-value < 0; ** when p-value < 0.001; * when p-value < 0.05; . when p-value < 0.1.

|  | **Model** | **Fixed effects** | **Terms** | **Estimate** | **Std. error** | **df** | **t value** | **Pr(>\|t\|)** |  |
| --- | --- | --- | --- | --- | --- | --- | --- | --- | --- |
| **DML** | GLMM, family=Gaussian AIC=-280.013 | Mantle length~pH+(1\|Replicate) | (Intercept) | 0.767 | 0.016 | 2.964 | 46.870 | < 0.001 | *** |
|  |  |  | pH | -0.019 | 0.023 | 3.054 | -0.830 | 0.466 |  |
| **Weight** | GLMM, family=Gaussian AIC=-587.700 | Weight~pH+(1\|Hatching Date) | (Intercept) | 9.50×10^-02^ | 5.49×10^-03^ | 1.11×10^0^ | 17.300 | 0.028 | * |
|  |  |  | pH | 1.72×10^-03^ | 2.56×10^-03^ | 1.03×10^2^ | 0.671 | 0.504 |  |

**Supplemental Table 3.** Results of the statistical models applied for analysis of ocean acidification effects in *Sepia officinalis*. Codes: *** when p-value < 0; ** when p-value < 0.001; * when p-value < 0.05; . when p-value < 0.1.

|  | **Model** | **Fixed effects** | **Terms** | **Estimate** | **Std. error** | **t value** | **Pr(>\|t\|)** |  |
| --- | --- | --- | --- | --- | --- | --- | --- | --- |
| **TBL** | GLM, family=Gaussian AIC=-200.786 | Total length~pH | (Intercept) | 1.139 | 0.012 | 92.580 | < 0.001 | *** |
|  |  |  | pH | -0.018 | 0.018 | -1.025 | 0.308 |  |
| **Reaction time** | GLM, family=Gamma, link=inverse AIC=342.260 | Reaction~pH | (Intercept) | 0.005 | 0.001 | 3.056 | 0.005 | ** |
|  |  |  | pH | 0.001 | 0.002 | 0.416 | 0.681 |  |
| **Catch time** | GLM, family=Gamma, link=inverse AIC=203.310 | Catch~pH | (Intercept) | 0.060 | 0.021 | 2.887 | 0.008 | ** |
|  |  |  | pH | 0.023 | 0.036 | 0.629 | 0.535 |  |

**Supplemental Table 4.** Results of the statistical models applied for analysis of ocean acidification effects in *Sepia officinalis*. Codes: *** when p-value < 0; ** when p-value < 0.001; * when p-value < 0.05; . when p-value < 0.1.

|  | **Model** | **Fixed effects** | **Terms** | **Estimate** | **Std. error** | **t value** | **Pr(>\|z\|)** |  |
| --- | --- | --- | --- | --- | --- | --- | --- | --- |
| **Fulton's index** | GLMM, family=Gamma, link=log AIC=650.300 | Fulton Index ~pH + (1\|Replicate) + (1\|Hatching Date) | (Intercept) | 3.006 | 0.180 | 16.686 | < 0.001 | *** |
|  |  |  | pH | 0.097 | 0.130 | 0.744 | 0.457 |  |
